# Proton Sharing in Polycarboxylic Acids in Aqueous Solution

**DOI:** 10.1021/jacsau.6c00377

**Published:** 2026-05-11

**Authors:** Lukáš Tomaník, Jiří Tůma, Gunnar Öhrwall, Ronny Golnak, Ngoc Lan Le Nguyen, Bruno Credidio, Harmanjot Kaur, Petr Slavíček, Bernd Winter, H. Christian Schewe

**Affiliations:** † Department of Physical Chemistry, 52735 University of Chemistry and Technology, Prague, Technická 5, Prague 16628, Czech Republic; ‡ Department of Molecular Physics, 28259 Fritz Haber Institute of the Max Planck Society, Faradayweg 4-6, Berlin 14195, Germany; § Department of Organic Chemistry, 52735 University of Chemistry and Technology, Prague, Technická 5, Prague 16628, Czech Republic; ∥ MAX IV Laboratory, Lund University, Lund 22100, Sweden; ⊥ Department of Highly Sensitive X-Ray Spectroscopy, 28340 Helmholtz-Zentrum Berlin für Materialien und Energie, Berlin 14109, Germany; # Institute of Organic Chemistry and Biochemistry of the Czech Academy of Sciences, Flemingovo nám. 542, Prague 160 00, Czech Republic; ∇ Department of Physics, Freie Universität Berlin, Arnimallee 14, Berlin 14195, Germany; ○ 89220 J. Heyrovský Institute of Physical Chemistry, Czech Academy of Sciences, Dolejškova 3, Prague 18223, Czech Republic

**Keywords:** Proton Sharing, Citric Acid (Krebs) Cycle, Photoelectron Spectroscopy, Biomolecules, Carboxylic Acids

## Abstract

We investigate proton sharing in biologically relevant polycarboxylic acids in aqueous solutions using liquid-jet X-ray photoelectron spectroscopy. In contrast to techniques that record time-averaged proton positions, like nuclear magnetic resonance, photoelectron spectroscopy captures instantaneous proton distribution due to its ultrafast probing time scale. The method is, therefore, uniquely sensitive to the occurrence of proton sharing in an aqueous environment, as demonstrated here. We only observe significant proton sharing dynamics in maleic acid, where its monoanionic form shows a single, delocalized peak in the carbon 1s spectrum. Conversely, succinic acid, fumaric acid, malic acid, glutaric acid, and citric acid exhibit distinct peaks corresponding to separate, localized COOH and COO^–^ groups, instead of shared protons. Our results reveal that intramolecular proton sharing in water is rather an exception for most biologically relevant polycarboxylic acids. This highlights the importance of enzyme-driven structural changes of polycarboxylic acids associated with proton sharing during metabolic processes.

## Graphical abstract

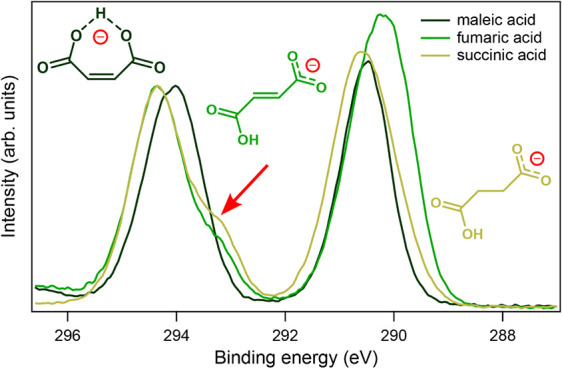

## Introduction

Proton sharing (PS), particularly in short, strong hydrogen bonds (SSHB), results from quantum mechanical effects. The low mass of the proton enables quantum phenomena such as proton tunneling, zero-point energy, and delocalization to exert a substantial influence on dynamics at room temperature.^1,2^ In this work, we define PS as a proton being bound to two molecular sites most of the time, rather than being localized on a single site.^3^ In more detail, PS can be defined in geometric terms, in line with the common SSHB classification: A proton is shared between two neighboring heteroatoms of, for example, two adjacent carboxylic groups (−COO^–^), when the O··O distance between the two oxygen atoms (from the two carboxylic groups) is less than 2.7 Å.^4–6^ Conversely, a proton is localized only on a single group if the O··O distance between the two oxygen atoms from the two carboxylic groups is greater than 2.7 Å. In this work (as detailed below), PS in monoanions is indicated when the two carboxyl groups are instantaneously equivalent on the photoelectron-spectroscopy time scale. This results in a single symmetric carbon 1s spectral feature, whereas a localized proton yields two distinct COOH and COO^–^ components. Exploring the extent of PS is crucial in understanding how enzymes utilize proton delocalization between hydrogen bonds and networks of these bonds to stabilize intermediates and transition states during chemical reactions.^7^ Proton delocalization controls a wide range of catalyzed enzymatic reactions where proton transfer steps are involved at oxygen or other heteroatoms, such as in racemization, aldol condensation, elimination, or even in photosynthesis.^8^ This delocalization can, for example, significantly alter the acidity (or basicity) of biomolecules, influencing the mechanism or the rate of the reaction.^2,9^

PS is essential to the citric acid cycle, also called the Krebs cycle, which is a central metabolic pathway. It plays a key role in energy metabolism, ultimately leading to the generation of Adenosine Triphosphate (ATP) from carbohydrates, fats, and proteins. Moreover, it dictates the biosynthesis by providing building blocks for many important metabolic processes. The reactions of the citric acid cycle are regulated by various enzymes and metabolites and can be adapted to the needs of the cell.^10^ Citric acid is a triprotic acid, containing three carboxyl (−COOH) groups, each capable of donating and sharing protons (H^+^). PS in aqueous enzymatic environments makes citric acid an excellent proton buffer and relay molecule. The presence or absence of PS is determined by the details of the associated microenvironment. A complete deprotonation scheme of citric acid in pH-adjusted water has been reported in an early study using Nuclear Magnetic Resonance (NMR) spectroscopy.^11^ The study concluded that remaining protons in singly and doubly deprotonated forms are localized on individual COOH groups rather than shared, as shown in Figure 1a, left branch. However, a subsequent study by different authors reevaluated the NMR data and concluded that this would imply nonphysically large interaction constants.^12^ Supported by ab initio calculations, they proposed a shared-proton model, as shown in Figure 1a, right branch.

**Figure 1:**
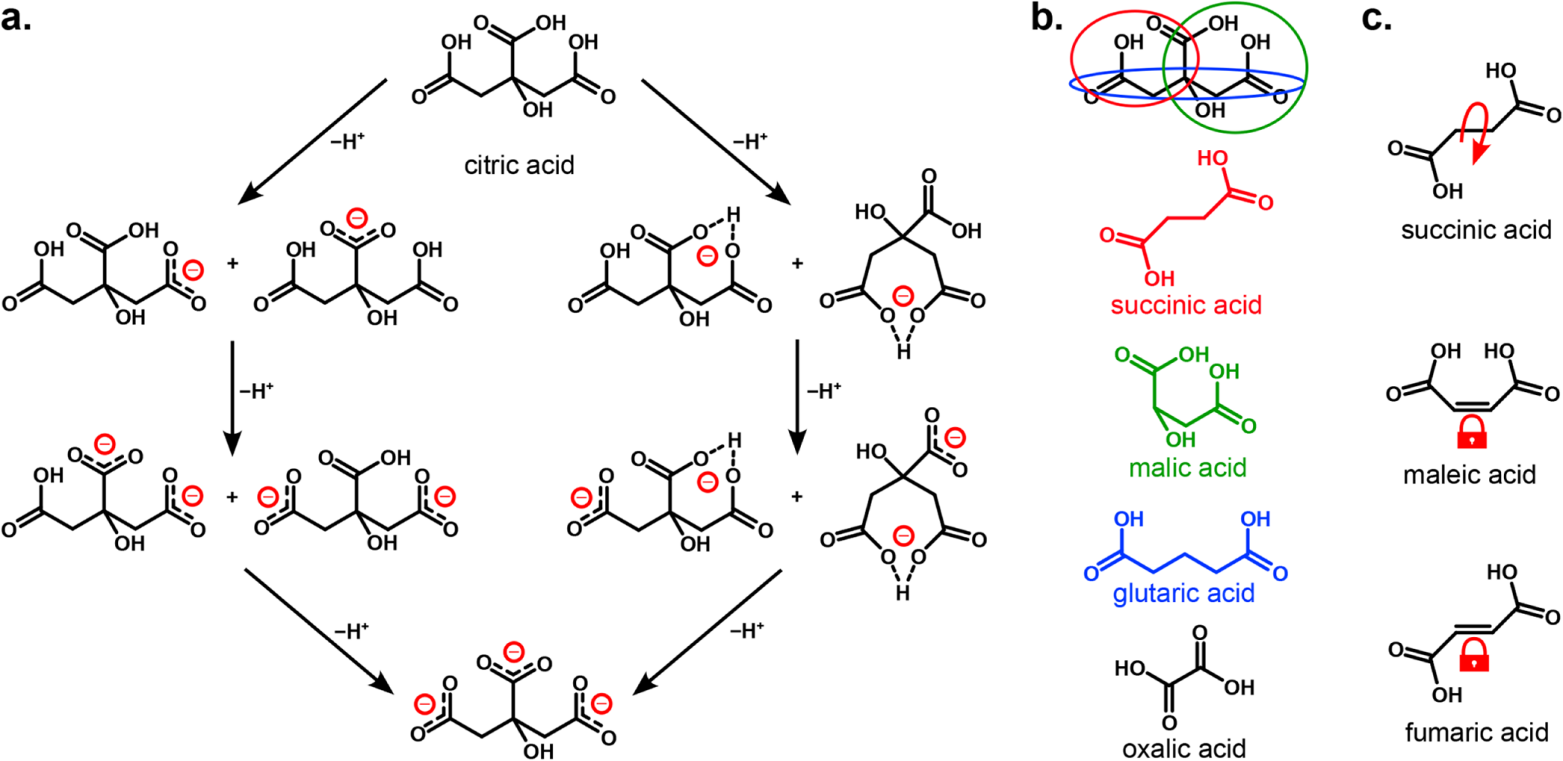
(**a**) Citric acid deprotonation scheme. The left branch shows the proton-localized model, and the right branch shows the proton-shared model. (**b**) Saturated dicarboxylic molecules investigated in this work. Fragments of citric acid are highlighted in colors. (**c**) Unsaturated C=C bond dicarboxylic molecules explored in this work.

PS has been investigated in a number of experimental and theoretical studies where it is sometimes also referred to as ″Low-Barrier Hydrogen Bonds″.^3^ In the gas phase, several studies used pulsed molecular beam Fourier transform microwave spectroscopy to study PS in carboxylic acid dimers.^13–18^ Computational studies revealed PS in the gas-phase maleate (singly deprotonated maleic acid)^19,20^ and malonaldehyde.^21^ In solids, X-ray and neutron diffraction or NMR are used to probe PS, e.g., in succinate (succinic acid monoanion) crystals^22^ or pressurized ice.^23^ Studies in solution usually use NMR or vibrational (infrared or Raman) spectroscopies. For example, they confirm the PS (sometimes referred to as ″Strong Intramolecular Hydrogen Bonds″ or ″Double-Well Proton Potential″) in protonated water Zundel ions^24^ or in maleate in various aprotic organic solvents.^25,26^ Succinic acid is also reported to exhibit some degree of PS in aprotic solvents, evidenced by NMR. This suggests a double-well potential for protons in its monoanion, where it is less pronounced than in maleic acid.^26^

To investigate PS in polyprotic acids in aqueous solution, we isolated the possible effect using a set of molecules (in addition to citric acid) containing only two carboxylic groups. In bulk water, carboxylic acids are expected to be predominantly monomeric due to strong hydration. Succinic acid, malic acid, and glutaric acid represent fragments of citric acid, as shown in Figure 1b, and are also active metabolites of the citric acid cycle. We also include the smallest possible dicarboxylic acid—oxalic acid. Moreover, we directly explore one specific geometric effect arising from locking the *cis* and *trans* configuration by introducing the C=C double bond on a four-carbon dicarboxylic acid by utilizing maleic acid and fumaric acid, which are also members of the citric acid cycle, shown in Figure 1c. Although introducing the C=C double bond can alter the electronic structure and ultimately influence the PS behavior, it provides clear geometric constraints. While fumaric acid is prohibited from any intramolecular PS between two COOH groups due to unfavorable geometry, the possible PS is eased in the case of maleic acid, as the COOH groups are fixed close to each other. Geometrically, these two molecules represent limiting cases of succinic acid’s free conformational space.

In this study, we utilize soft X-ray photoelectron spectroscopy (PES) to explore PS in aqueous solution. In the last two decades, PES has become a powerful method for chemical analysis expanded to the realm of liquids, notably as liquid-jet (LJ-) PES.^27^ It enables (among others) photoemission measurements in water and volatile aqueous solutions. Similar to chemical analysis of solids and gases (known as Electron Spectroscopy for Chemical Analysis, ESCA), LJ-PES determines electron binding energies (eBEs), which are element-specific and site-specific within a molecule in solution. Moreover, eBE shifts reveal information about the chemical state, such as protonation or deprotonation, or changes in the vicinity of individual atomic species. Given our set of molecules, it is sufficient to focus on measuring carbon core-level photoelectron spectra. The oxygen core-level signal resulting from the carboxylic groups of the solutes is hidden by the signal originating from the water solvent molecules.

Assuming proton localization for monoanions, one would expect two distinct peaks in the PE spectra originating from COOH and COO^–^. Conversely, if the proton is shared, a single symmetric peak would appear in the spectrum. Here, we benefit from LJ-PES’ unique property to probe molecular structures on subfemtosecond/attosecond time scales, which is much faster compared to NMR or vibrational spectroscopy, that typically averages over microseconds. Because the emission of the photoelectron is faster than nuclear motion, the spectrum reflects the instantaneous nuclear configuration distribution (including any quantum delocalization) rather than a time-averaged hopping picture. The ultrafast nature of photoionization has been utilized, for example, to resolve pseudoequivalent imidazole nitrogen atoms, which appear equivalent in NMR.^28^ Therefore, our results provide information on the eBEs associated with the instantaneous proton distribution rather than its time-averaged position (as provided by NMR).

## Results and Discussion

We recorded C 1s photoelectron spectra of aqueous solutions of the molecules shown in Figure 1 at different pH, preparing molecules in different (de)­protonation states. Experimental details, including solution pH and concentration, and p*K*_*a*_ values, can be found in the (Supporting Information SI). To begin with, Figure 2a shows spectra of maleic acid, fumaric acid, and succinic acid at low pH, where all carboxylic groups are protonated. The peak at ∼290.5 eV originates from aliphatic carbons (CH and CH_2_) while the single peak at ∼294.2 eV results from the two COOH groups, revealing a similar spectral feature for both COOH groups in a molecule (Table 1). In the case of fully protonated molecules, the possible spectral feature of PS is much more subtle, and the interpretation is more difficult. Therefore, we support the data by our quantum-chemical calculations using an efficient computational protocol,^29^ with details given in the SI. Comparing measured and calculated peak positions, the computational results summarized in Table S4 in the Supporting Information reveal that while succinic acid and fumaric acid do not manifest PS, maleic acid can exhibit PS, as shown in Figure 2a. Notably, the calculations reproduce even the small shifts in peak positions. Next, Figure 2b shows spectra of molecules in their monoionic forms, where PS is most likely to occur. Fumaric acid and succinic acid exhibit a double peak at ∼294 eV (highlighted by an arrow). These two peaks, separated by ∼1.15 eV (Table 1), originate from the respective COOH and COO^–^ groups, which is confirmed by our calculations (Table S4). This reveals a prevailing localized-proton model. Moreover, our supplementary calculations for succinic acid (Table S5) reveal a shallow double-well potential, suggesting that even if the proton is localized most of the time, it can oscillate between the two carboxyl groups. In contrast, maleic acid exhibits a single, symmetric peak at ∼294 eV, pointing to a significant PS, which is also in agreement with our calculations (Table S4). Finally, Figure 2c shows spectra of fully deprotonated molecules (measured at high pH), where PS is no longer possible. The peak at ∼293.3 eV results from COO^–^ groups, and agrees well with our calculations (Table S4).

**Figure 2:**
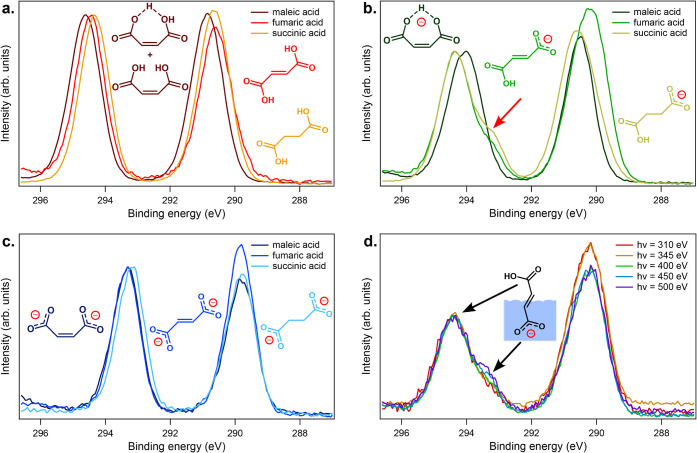
C 1s photoelectron spectra of maleic acid, fumaric acid, and succinic acid in pH-adjusted aqueous solutions (see Methods in SI for details) and their concluded molecular structures. (**a**) Fully protonated molecules at low pH. (**b**) Singly deprotonated molecules (monoanions) at middle pH. (**c**) Doubly deprotonated molecules at high pH. (**d**) Spectra of singly deprotonated fumaric acid measured with different photon energies.

**Table 1:** Peak Positions (eV) Extracted from C 1s Photoelectron Spectra of Investigated Molecules in pH-Adjusted Aqueous Solutions^a^

		Succinic acid	Maleic acid	Fumaric acid	Oxalic acid	Malic acid	Glutaric acid	Citric acid
Low pH	CH_2_/CH	290.65	290.86	290.59		290.65	290.24	290.66
	C–OH					292.10		292.27
	COOH	294.33	294.63	294.47	294.74	294.40	294.14	294.38
Middle pH	CH_2_/CH	290.56	290.52	290.25		290.28	290.33	290.48
	C–OH					291.60		291.99
	COO^–^	293.21		293.19		293.14	293.16	293.17
	COOHOOC		294.07					
	COOH + COO^–^				293.99			
	COOH	294.35		294.35		294.23	294.28	294.30
Higher pH	CH_2_							289.85
	C–OH							291.43
	COO^–^							293.08^b^
	COOH							293.66^b^
Highest pH	CH_2_/CH	289.83	289.82	289.84		289.86	289.75	289.76
	C–OH					291.31		291.35
	COO^–^	293.22	293.36	293.36	293.22	293.23	293.20	293.16

a Corresponding peak widths are summarized in Table S1 in the Supporting Information.

b High fitting uncertainty due to large peak overlap.

We now return to the fumaric acid and succinic acid spectra in Figure 2b. Considering pH and p*K*_*a*_ values, an equivalent number of COOH and COO^–^ groups is expected. Despite this, the COOH peak (∼294.4 eV) is significantly more intense than the COO^–^ peak (∼293.2 eV). Apart from possible differences in cross sections, the reason is that LJ-PES (unlike NMR or vibrational spectroscopy) is a surface-sensitive method. We recorded spectra for fumaric acid using different incident photon energies shown in Figure 2d, where higher photon energies result in a larger probing depth. The relative intensity of the COO^–^ peak increases with increasing probing depth, evidencing that this charged group is, on average, embedded deeper in the bulk solution. The COOH group provides a stronger signal since it resides on average closer to or directly at the surface.^30^

To further support our conclusions about PS and to highlight the unique ability of LJ-PES, we performed complementary ^1^H NMR titration measurements for succinic acid, fumaric acid, and maleic acid. The results, as well as a detailed discussion, are presented in the SI, while the two main conclusions from our NMR data are the following: (1) Due to a longer time scale in NMR, the proton exchange is inevitably included in the data, resulting in a single average peak covering both COOH and COO^–^ contributions. (2) We find a different trend in the measured chemical shifts of maleic acid compared to fumaric acid and succinic acid during titration. This is in agreement with our observations from LJ-PES (PS in maleic acid versus no PS in fumaric acid and succinic acid), although NMR cannot provide direct evidence of PS. This leads us to the conclusion that LJ-PES provides a truly unique direct probe of PS that other methods cannot provide.

We now discuss potential PS in citric acid and the other set of its intermediates: malic acid, glutaric acid, together with the smallest dicarboxylic acid—oxalic acid. Their C 1s photoelectron spectra from pH-adjusted aqueous solutions are presented in Figure 3, and the extracted peak positions are summarized in Table 1. Figure 3a shows spectra of oxalic acid. While a single peak at low and high pH corresponds to COOH and COO^–^, respectively, a single peak obtained for the singly protonated molecule should not be taken as evidence for PS. It is rather an effect of the eBE changes in the two groups, causing these peaks to move together. This interpretation is based on the following: (i) Our theoretical modeling shows that due to the molecular geometry, the distance between the two oxygen atoms from the two carboxylic groups is too large in water for PS. (ii) The peak for medium pH is significantly broader than for low/high pH (σ of 0.7 vs 0.5 eV; Table S1 in the Supporting Information), suggesting two overlapping peaks. (iii) Our calculations (Table S4) suggest a slightly smaller peak separation for oxalic acid than for fumaric acid or succinic acid, which can result in the peak overlap in oxalic acid. Figure 3b presents spectra of malic acid. At low pH, the peaks at ∼294.4 eV, ∼292.1 eV, and ∼290.7 eV originate from COOH, C–OH, and CH_2_, respectively. At middle pH, these peaks are shifted to lower BEs, and a new peak from COO^–^ emerges. At high pH, the peaks are further shifted to lower BEs, and the COOH peak disappears. The presence of both COOH and COO^–^ peaks at middle pH reveals that protons are localized on single groups. A similar conclusion is drawn from the glutaric acid spectra shown in Figure 3c. While the peak at ∼290 eV comes from CH_2_, peaks at ∼294 eV and ∼293 eV originate from COOH and COO^–^. As the spectrum from the middle-pH solution exhibits both peaks, it points to localized protons rather than PS. Finally, spectra of citric acid are shown in Figure 3d. For the lowest pH (0.8), we observe three peaks at 290.7, 292.3, and 294.4 eV, originating from CH_2_, C–OH, and COOH, respectively. For a higher pH of 3.9, a new COO^–^ peak at 293.2 eV appears in addition to the previous three ones. This suggests a prevailing proton-localized arrangement. For a pH of 5.6, the COOH peak decreases in intensity and moves to lower BE (∼293.7 eV), forming a shoulder to the COO^–^ peak, as the molecule gets an overall double negative charge. This result again suggests a proton-localized arrangement. At the highest pH of 10.9, the COOH peak disappears, adding to the COO^–^ peak intensity as all carboxylic groups in the molecule are deprotonated at this pH.

**Figure 3:**
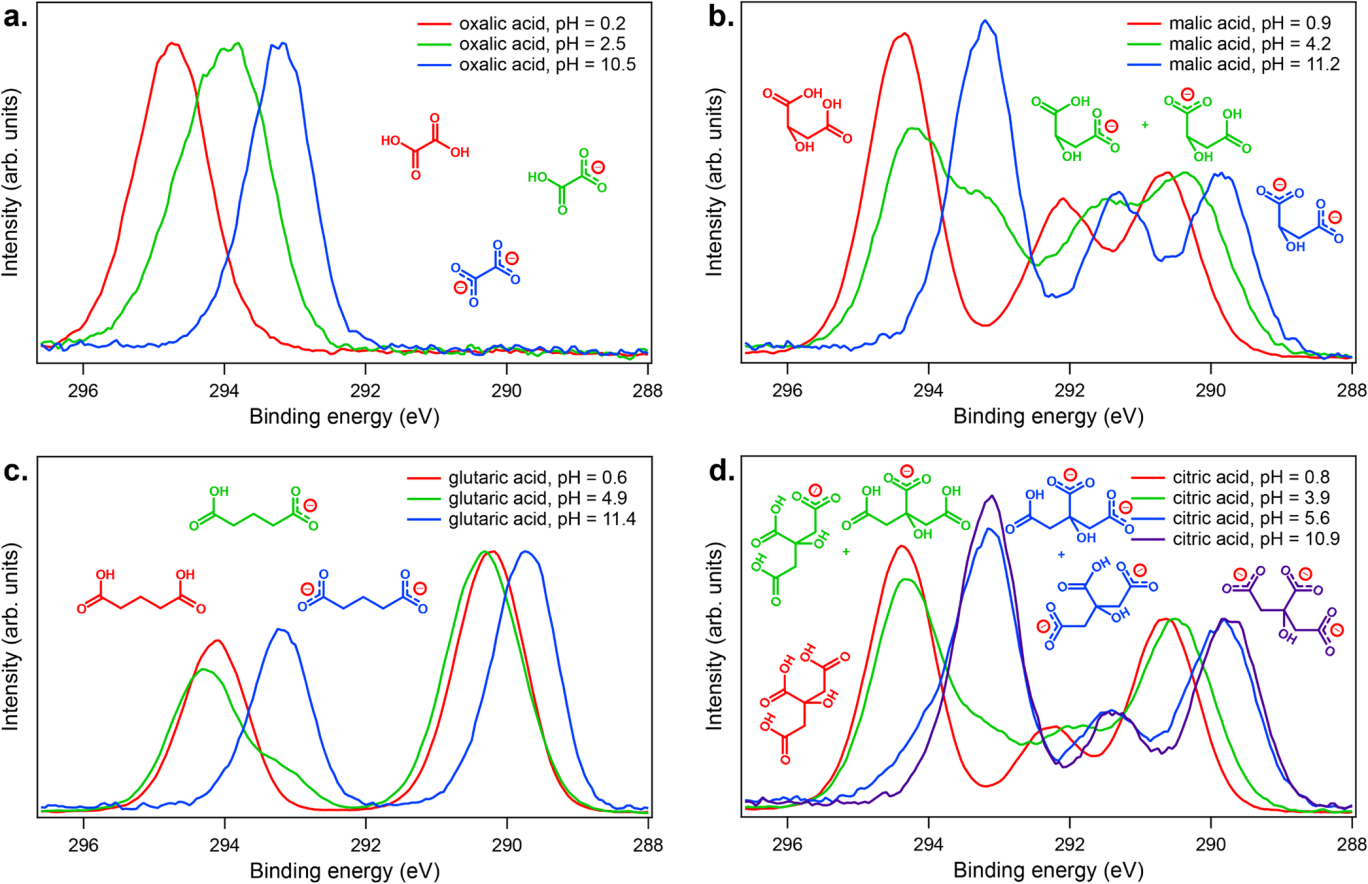
C 1s photoelectron spectra measured in pH-adjusted aqueous solutions of (**a**) oxalic acid, (**b**) malic acid, (**c**) glutaric acid, and (**d**) citric acid. Concluded molecular structures for each protonation state are presented.

We conclude that a proton-localized arrangement has been identified in citric acid in water for both the singly (pH of 3.9) and doubly (pH of 5.6) deprotonated cases. However, this result does not rule out the possibility of PS occurring in citric acid in general. It merely results from the conformationally unrestricted structures of citric acid in water. As our simulations show, the average distance between the oxygen atoms of two neighboring carboxylic groups is much larger than 2.7 Å which prohibits PS.

## Conclusions

We investigated the potential for PS in various biologically relevant polycarboxylic acids in aqueous solution. We report a significant PS in maleic acid. In contrast, we found that protons are mostly localized (rather than shared) on single carboxylic groups in aqueous solutions of succinic acid, fumaric acid, oxalic acid, malic acid, glutaric acid, and citric acid. This provides strong evidence that intramolecular proton sharing in Krebs-cycle-intermediate, polycarboxylic acids such as citric acid, when hydrated in water, is not a prevailing phenomenon. Thus, it advocates for the proton-localized model (left branch of Figure 1a) over the proton-shared model (right branch of Figure 1a). Any degree of flexibility that allows the molecules to adopt a stable conformation beyond the close proximity of the carboxylic groups dictates the prevalence of localized protonation rather than PS. This is consistent with the widely held view that carboxylic groups form strong hydrogen bonds with water molecules.

However, when present in biological systems, carboxylic acids are found to interact with proteins in fixed conformations. In other words, the coordination of a carboxylic acid inside an active site of an enzyme within the Krebs cycle may well facilitate PS, particularly if the geometric conditions of SSHB are met. We demonstrated that the utilized method of LJ-PES is an effective probe to investigate PS. Given the element- and site-specific sensitivity of LJ-PES, there is no fundamental limit to its use in probing PS at enzyme-active sites. This is, however, beyond the scope of the present investigation. Moreover, LJ-PES enables us to probe the electronic structure on ultrafast time scales, thus revealing the immediate proton position rather than its averaged position. This facilitates direct investigation of associated nuclear quantum effects^31^ while concomitantly establishing a framework for the study of proton sharing phenomena in a variety of chemical systems and applications. For example, proton delocalization is believed to be the mechanism behind non-nucleophilic organic superbases^32^ and proton sponges^33^ or the high electrical conductivity in eutectic solvents.^34^

## Supplementary Material



## Notes

Experimental details on LJ-PES measurements; core-level LJ-PES spectra calculations; comparison of different experimental methods to probe the effect of proton sharing (PDF)
