# Supplementary material for: Proton Sharing in Polycarboxylic Acids in Aqueous Solution
Source: JACS Au. 2026 May 11;6(6):3349–55. doi: 10.1021/jacsau.6c00377 (PMC13291952; doi:10.1021/jacsau.6c00377)
Supplement: Supplementary file 1 [file au6c00377_si_001.pdf]

Supplementary Information: Proton Sharing in Polycarboxylic Acids in Aqueous Solution

Lukáš Tomaník,[*,†,‡] Jiří Tůma,[¶] Gunnar Öhrwall,[§] Ronny Golnak,[‖] Ngoc Lan Le Nguyen,[⊥] Bruno Credidio,[‡] Harmanjot Kaur,[‡,#] Petr Slavíček,[†] Bernd Winter,[‡] and H. Christian Schewe[*,@,⊥]

†*Department of Physical Chemistry, University of Chemistry and Technology Prague, Technická 5, 16628 Prague, Czech Republic*

‡*Department of Molecular Physics, Fritz Haber Institute of the Max Planck Society, Faradayweg 4-6, 14195 Berlin, Germany*

¶*Department of Organic Chemistry, University of Chemistry and Technology Prague, Technická 5, 16628 Prague, Czech Republic*

§*MAX IV Laboratory, Lund University, 22100 Lund, Sweden*

‖*Department of Highly Sensitive X-Ray Spectroscopy, Helmholtz-Zentrum Berlin für Materialien und Energie, 14109 Berlin, Germany*

⊥*Institute of Organic Chemistry and Biochemistry of the Czech Academy of Sciences, Flemingovo nám. 542, 160 00 Prague, Czech Republic*

#*Department of Physics, Freie Universität Berlin, Arnimallee 14, 14195 Berlin, Germany*

@*J. Heyrovský Institute of Physical Chemistry, Czech Academy of Sciences, Dolejškova 3, 18223 Prague, Czech Republic*

E-mail: tomanikl@vscht.cz; christian.schewe@jh-inst.cas.cz

1. Experimental details on LJ-PES measurements

Liquid-jet photoelectron spectra were measured at the soft X-ray beamline FlexPES,[1] at the MAX IV synchrotron facility (Lund, Sweden), using the beamline's liquid-jet setup. The photon energy of 400.3 eV was used, calibrated as follows. The C 1s photoelectron spectra of multiple samples were measured using the beamline's first-order and second-order light. Considering C 1s binding energies of roughly 290 eV, the photoelectrons emitted by the first-order light (∼400 eV) were recorded at ∼110 eV kinetic energy, excluding bias voltage (see below). The photoelectrons emitted by the second-order (∼800 eV) light were recorded at ∼510 eV kinetic energy, excluding bias voltage. The difference between the fitted kinetic-energy peak positions recorded with the second-order and the first-order light corresponds to the precise photon energy. The beamline vertical slit size was 100 µm, corresponding to a photon band width of ∼0.15 eV. A liquid microjet in vacuum was produced via a silica nozzle of an inner diameter of 25 μm. Solutions were delivered by a high-performance liquid chromatography (HPLC) pump at a constant flow rate of 0.6 mL/min. A voltage source was connected to the jet rod and the body of the setup to apply a bias voltage to the jet. The vacuum of $\sim 10^{-5}$ mbar in the interaction chamber was maintained using Roots fore-vacuum pumps and turbomolecular high-vacuum pumps. The injected liquid was collected in liquid-nitrogen-cooled traps at the far end of the interaction chamber. The laminar-flow part of the jet, typically extending 5–10 mm from the nozzle, was irradiated by the X-ray beam in a perpendicular orientation. Photoelectrons were detected in a perpendicular geometry, corresponding to an angle of 90° with respect to the light propagation and to the liquid jet. The detector was placed in a horizontal position to maximize the XPS signal, as the beamline provides horizontally polarized light. The detection of photoelectrons was done via a hemispherical analyzer. The analyzer slit of 0.5 mm and pass energy of 200 eV correspond to the analyzer resolution of 250 meV.

The photoelectron peaks were fitted with Gaussian functions and a linear background. The fitted peaks' widths are summarized in Table S1. The reported binding energies (BE) in

this work refer to the absolute energy scale (with respect to the vacuum) and were determined using a difference between the respective peak position (P) and the low-kinetic-energy cutoff (C), using precisely calibrated photon energy ($h\nu$), BE = $h\nu - (\text{P} - \text{C})$. We applied a bias voltage of −50 V to the liquid (accelerating the photoelectrons) in all measurements. This procedure of determining absolute BEs in liquids has been detailed in Ref.[2]

Table S1: Peak widths (eV) expressed as σ of the respective Gaussian fits pertaining to Table 1 in the main text.

		Succinic acid	Maleic acid	Fumaric acid	Oxalic acid	Malic acid	Glutaric acid	Citric acid
Low pH	CH_2/CH	0.49	0.48	0.49		0.47	0.49	0.47
	C–OH					0.49		0.47
	COOH	0.45	0.48	0.45	0.52	0.46	0.45	0.46
Medium pH	CH_2/CH	0.57	0.45	0.56		0.53	0.53	0.52
	C–OH					0.60		0.54
	COO^-	0.38		0.34		0.42	0.36	0.37
	COOHOOC		0.52					
	COOH+COO^-				0.66			
	COOH	0.49		0.50		0.48	0.47	0.49
Higher pH	CH_2							0.47
	C–OH							0.47
	COO^-							0.47*
	COOH							0.46*
Highest pH	CH_2/CH	0.45	0.45	0.42		0.44	0.45	0.45
	C–OH					0.46		0.42
	COO^-	0.42	0.43	0.42	0.45	0.44	0.41	0.43

* High fitting uncertainty due to large peak overlap.

Preparational experiments on the flow properties of the liquid jets depending on the solutions' pH and concentration, as well as pK_a values, were tested in our LJ-PES laboratory at the Department of Physical Chemistry of the University of Chemistry and Technology, Prague. 0.5M solutions of carboxylic acids were prepared by dissolving the respective amount of commercially available chemicals in MilliQ water (18.2 MΩ·cm). The pH was adjusted by adding HCl or hydroxide (NaOH, KOH, or LiOH, based on the product's solubility) and monitored by a pH meter. No unexpected or unusually high safety hazards were encountered. All solutions of the same compound prepared with different hydroxides provided highly reproducible spectra, confirming that the specific cation in the hydroxide compound does not influence the results. The properties of the prepared solutions are summarized in Table S2. The target pH of solutions was selected based on acidity constants (pK_a values), summarized in Table S3.

Table S2: Properties of aqueous solutions measured with LJ-PES.

Solution	pH	pH agent
Maleic acid	0.03	HCl
Maleic acid	4.05	KOH
Maleic acid	4.08	LiOH
Maleic acid	9.45	KOH
Fumaric acid	0.99	HCl
Fumaric acid	3.75	NaOH
Fumaric acid	11.65	NaOH
Succinic acid	1.09	HCl
Succinic acid	4.89	NaOH
Succinic acid	11.63	NaOH
Glutaric acid	0.58	HCl
Glutaric acid	4.90	NaOH
Glutaric acid	11.38	NaOH
Oxalic acid	0.15	HCl
Oxalic acid	2.48	LiOH
Oxalic acid	10.52	LiOH
Malic acid	0.89	HCl
Malic acid	4.20	NaOH
Malic acid	11.20	NaOH
Citric acid	0.78	HCl
Citric acid	3.89	NaOH
Citric acid	5.62	NaOH
Citric acid	10.92	NaOH

Table S3: Acidity constants (pK_a) of compounds used in this work, taken from ref.[3]

Compound	pK_{a1}	pK_{a2}	pK_{a3}
Maleic acid	1.92	6.23	
Fumaric acid	3.02	4.38	
Succinic acid	4.21	5.64	
Glutaric acid	4.32	5.42	
Oxalic acid	1.25	3.81	
Malic acid	3.40	5.11	
Citric acid	3.13	4.76	6.40

2. Core-level LJ-PES spectra calculations

Peak positions were calculated using our efficient computational protocol.[4] The protocol is based on a single optimized structure employing a hybrid explicit-implicit solvation, where the number of explicit solvent molecules increases with the increasing negative charge of the solute. Specifically, we used 0, 6, and 12 explicit water molecules for protonated, singly

deprotonated, and doubly deprotonated molecules, respectively, for maleic, fumaric, and succinic acids. For oxalic acid, we used 4, 10, and 12 water molecules, respectively, to overcome the convergence issues. We used the polarizable continuum model[5,6] (PCM) on top of the explicit solvation. Universal force field (UFF) atomic radii[7] and an electrostatic scaling factor $\alpha = 1.1$ parameters were used for PCM. The structures were optimized using Gaussian 09, revision D.01.,[8] employing the hybrid functional based on the B3LYP functional with the Coulomb-attenuating method, together denoted as CAM-B3LYP,[9] and Pople's basis set 6-31+G*.

Core-level calculations were done using the maximum-overlap method,[10] the CAM-B3LYP functional, the aug-cc-pVTZ basis set[11,12] for hydrogen and oxygen atoms, and the core-enhanced aug-cc-pCVTZ basis set[13] for carbon atoms. Although using basis sets optimized specifically for core-level spectroscopies was shown to be very effective,[14] we base our choice of the CAM-B3LYP/aug-cc-pCVTZ combination on our own testing and experience with the great performance of the approach.[4] The solvent effects were included by non-equilibrium PCM.[15,16] The calculations were performed in Q-Chem, version 6.0.[17]

We show the optimized structures in Figures S1, S2, S3, and S4. The calculated peak positions assuming either proton-shared or proton-localized model are summarized in Table S4.

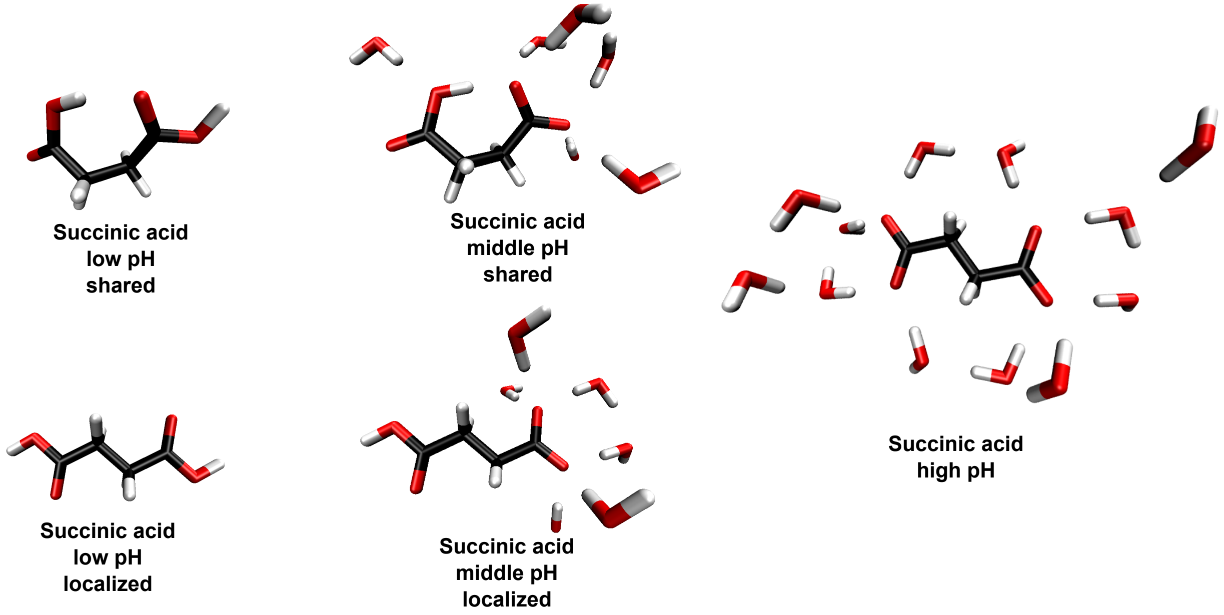

Figure S1: Optimized structures of succinic acid used for calculations of C 1s LJ-PES peak positions.

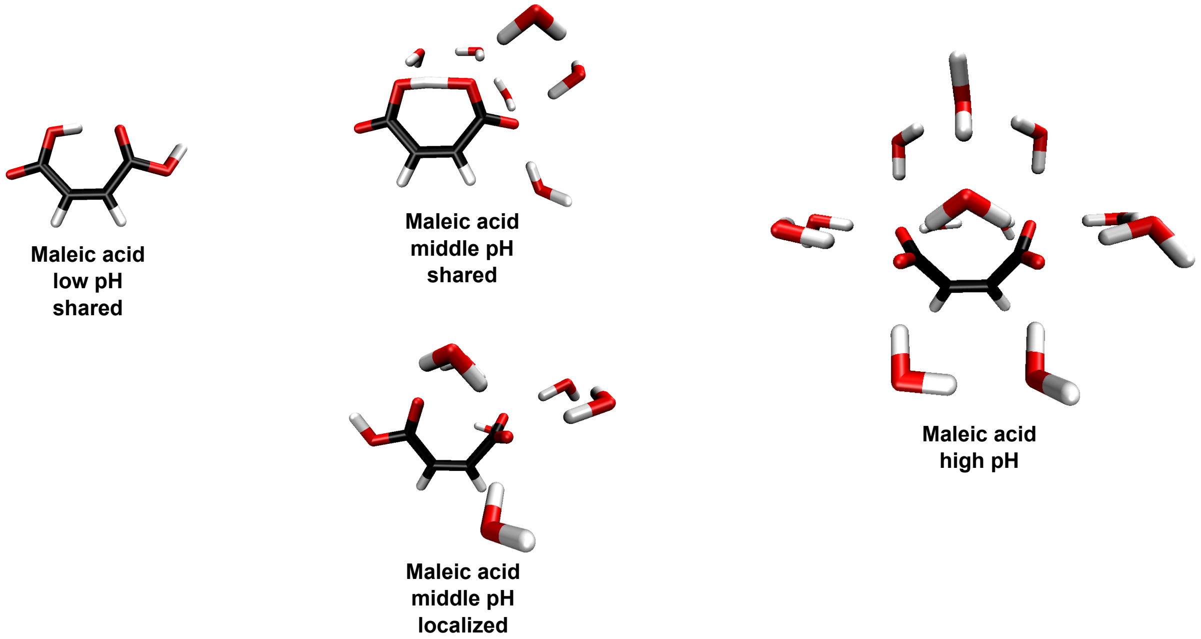

Figure S2: Optimized structures of maleic acid used for calculations of C 1s LJ-PES peak positions.

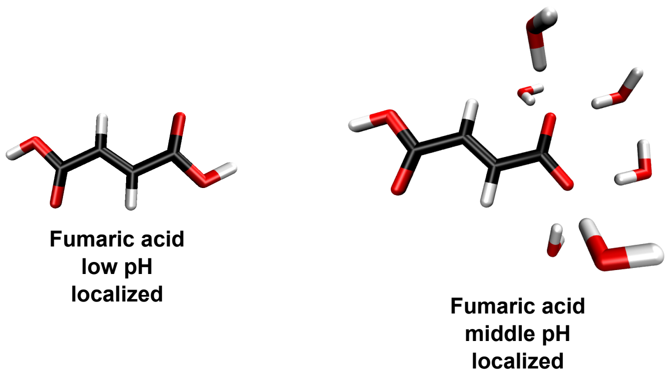

Figure S3: Optimized structures of fumaric acid used for calculations of C 1s LJ-PES peak positions.

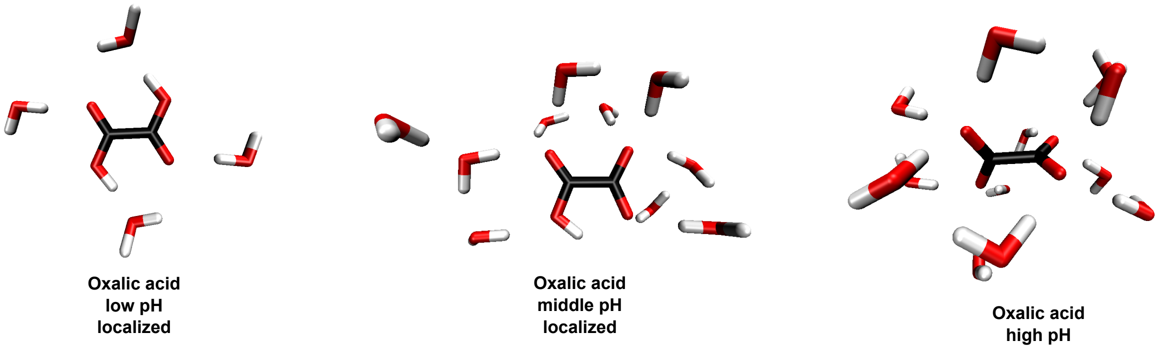

Figure S4: Optimized structures of oxalic acid used for calculations of C 1s LJ-PES peak positions.

3. Potential-energy-surface calculations

To determine the energy barrier between proton localization on one carboxyl group and the other in singly deprotonated succinic acid, we performed a relaxed scan calculation in Gaussian 09, revision D.01.[8] Specifically, we used the CAM-B3LYP functional with 6-31+g* basis set and PCM to model the solvation. Note, however, that no explicit water molecules were included in the scan calculations. The proton was moved from one carboxyl to another with a step size of 0.02 Å. In each step, the geometry of all other atoms was relaxed to its minimum. The energies are summarized in Table S5. The difference between the first (fully optimized) point and the maximum energy was 0.02 eV. Considering two lower states (proton localization on one or the other carboxyl) and one intermediate state (shared proton), the

Table S4: Calculated peak positions (eV) in photoelectron spectra of investigated molecules in pH-adjusted aqueous solutions. "Shared" and "localized" categories show results assuming hypothetical proton sharing and proton localization, respectively.

	Succinic acid		Maleic acid		Fumaric acid	Oxalic acid
	shared	localized	shared	localized	localized	localized
Low pH	290.61 (CH_2)	*	290.80 (CH)		290.58 (CH)	
	294.48 (294.83+294.13) (COOHOOC)	294.38 (COOH)	294.68 (294.98+294.37) (COOHOOC)		294.49 (COOH)	295.01 (COOH)
Medium pH	290.16 (CH_2)	290.01 (CH_2)	290.27 (CH)	290.20 (CH)	290.15 (CH)	
	293.79 (293.97+293.60) (COOHOOC)	293.38 (COO^-)	293.96 (294.11+293.81) (COOHOOC)	293.66 (COO^-)	293.57 (COO^-)	294.15 (COO^-)
		294.23 (COOH)		294.36 (COOH)	294.28 (COOH)	294.81 (COOH)
High pH	*		289.62 (CH)		289.70 (CH)	
	*		293.43 (COO^-)		293.35 (COO^-)	293.40 (COO^-)

[*] Data missing due to failing to converge the core-level ionization calculations.

Boltzmann distribution yields approximately 80 : 20 for localized : shared proton. It is important to note that this reflects only the distribution in the geometrically favorable carbon-skeleton conformation, which allows proton sharing, and that other (geometrically unfavorable) conformations are also present (see Figure S1, middle part). Consequently, the total distribution of proton-shared structures is expected to be significantly lower than 20 %.

Table S5: Optimized energies (Hartree) for the individual steps of relaxed scan of proton localization in aqueous succinic acid.

Step number	Energy
1	-456.38069
2	-456.38067
3	-456.38064
4	-456.38058
5	-456.38051
6	-456.38042
7	-456.38032
8	-456.38022
9	-456.38013
10	-456.38004
11	-456.37998
12	-456.37995
13	-456.37995
14	-456.38000
15	-456.38009
16	-456.38023
17	-456.38040
18	-456.38056
19	-456.38069

4. Comparison of different experimental methods to probe the effect of proton sharing

In the following, we discuss the capabilities to probe proton sharing (PS) in the liquid phase using different experimental methods compared to LJ-PES. As pointed out in the main text, pulsed-beam Fourier transform microwave spectroscopy has proven to be a powerful method to investigate PS in the gas phase, where carboxylic acid dimers exhibit PS dynamics in a concerted multiple proton transfer.[18–23] However, the technique is not suitable for investigating PS in the liquid phase. Apart from LJ-PES, other promising techniques comprise (1) Nuclear Magnetic Resonance (NMR), which may probe differences in the chemical shifts at the protonation sites upon proton delocalization, and (2) infrared and/or Raman spectroscopies, which may be sensitive to changes of vibrational modes at the functional groups where the proton is delocalized.

The main difference between these techniques is the probing time: while all four methods record signals comprising the incoherent summation of probing the dynamics within individual molecular entities in solution, the main difference stems from how long it takes to probe a particular interaction. LJ-PES probes molecular structures on sub-femtosecond/attosecond timescales defined by the photoionization process. Thus, PES is effectively probing "frozen" molecular geometry, as the processes of photon absorption and the subsequent electron emission are much faster than any nuclear motion. In contrast, NMR techniques or vibrational spectroscopies typically record signals by averaging over microseconds. However, it is important here to distinguish whether the microsecond timescales are mainly technical limitations or really the duration required to probe a particular process.

4.1 NMR titration

As an extension of our experimental work, we have studied the selected dicarboxylic acids (maleic acid – MA, fumaric acid – FA, and succinic acid – SA) by means of NMR as a

representative of traditional spectroscopic methods for probing organic compounds. 40 μmol of each acid was dissolved in excess of D_2O (ca 1.5 mL). Each sample was then studied by ^{1}H NMR spectroscopy (instrument: JEOL JNM-ECZL400G; working frequency 400 MHz) with emphasis on the change of chemical shift of the hydrocarbon backbone signal, i.e., CH=CH double bond signal for MA and FA, and CH_2–CH_2 signal for SA. Between each measurement, 8 μl of 1M KOH solution in D_2O was added, i.e., 8 μmol (0.2 equiv.) of KOH was inserted per addition. The general deprotonation scheme is shown in Figure S5. The achieved results are summarized in Figures S6, S7, and S8.

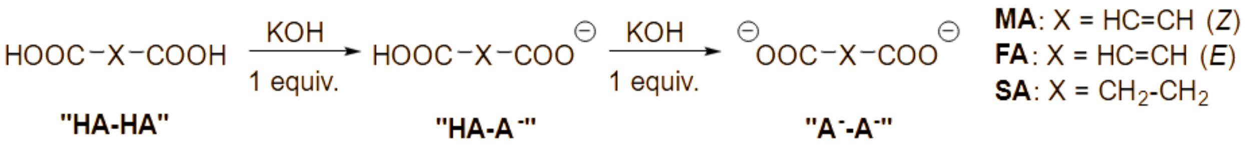

Figure S5: ^{1}H NMR titration scheme of MA, FA, and SA.

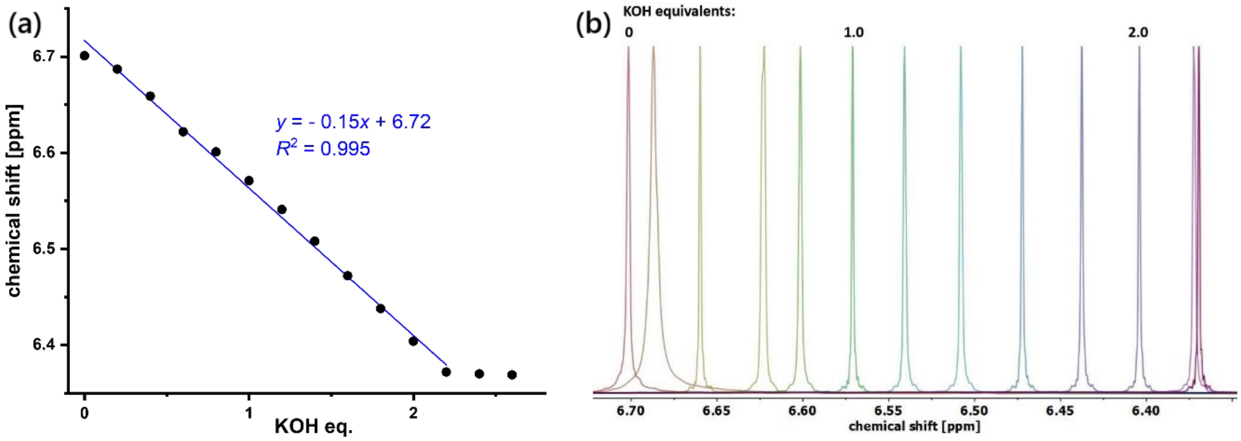

Figure S6: NMR titration of fumaric acid (FA): (a) chemical shift as a function of KOH equivalents; (b) progression of ^{1}H NMR peaks upon addition of 0.2 equivalent aliquots of KOH.

From the presence of only a single peak in the NMR spectrum, it is apparent that NMR cannot effectively distinguish between the various states formed of the native acid HA–HA, singly deprotonated acid HA–A^-, and doubly deprotonated acid A^-–A^- (Figure S5). The process of proton exchange between the various co-existing species is dramatically faster as opposed to the NMR scanning period that can typically resolve processes occurring on a

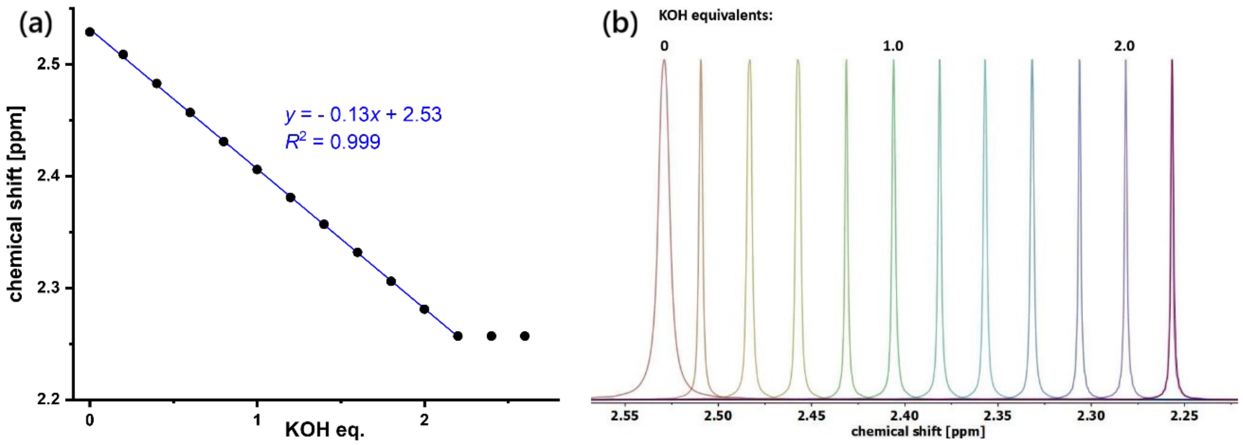

Figure S7: NMR Titration of succinic acid (SA): (a) chemical shift as a function of KOH equivalents; (b) progression of ^{1}H NMR peaks upon addition of 0.2 equivalent aliquots of KOH.

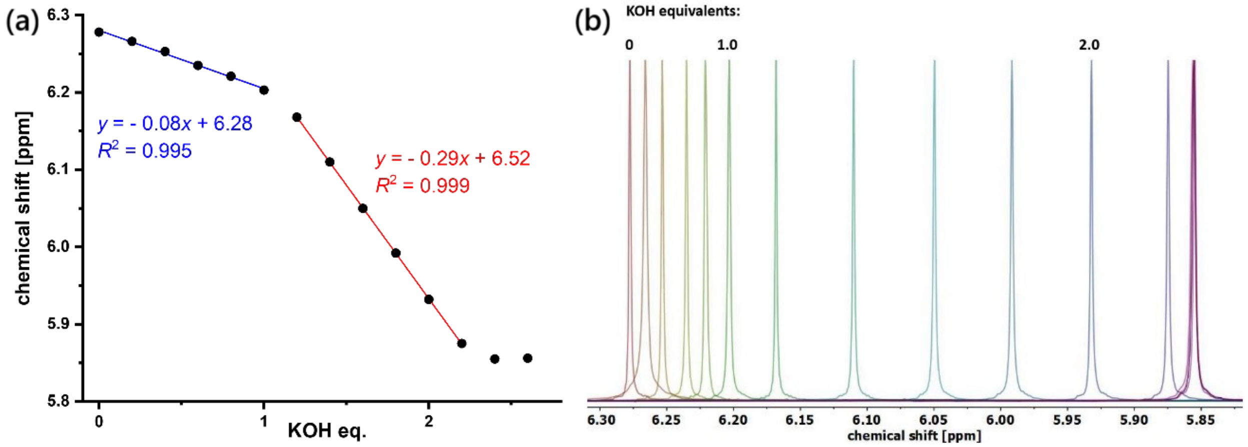

Figure S8: NMR Titration of maleic acid (MA): (a) chemical shift as a function of KOH equivalents; (b) progression of ^{1}H NMR peaks upon addition of 0.2 equivalent aliquots of KOH.

micro- to millisecond scale or slower. Therefore, the resulting chemical shift represents an average value of all the species present in the solution for each data point collected. For example, a data point upon the addition of 0.4 equiv. of KOH corresponds to the average chemical shift of 60% of HA–HA and 40% of HA–A$^-$. A chemical shift corresponding to 1.4 equiv. of KOH results from the presence of 60% of HA–A$^-$ and 40% of A$^-$–A$^-$. It is worth noting that in all three cases (MA, FA, SA), the saturation point of 100% concentration of A$^-$–A$^-$ was found upon addition of approximately 2.2 equiv. of KOH rather than 2.0 equiv.

This result likely stems from the air moisture absorbed by the solid KOH that was used for the preparation of the 1M KOH/D_2O solution utilized in the experiments. Since this experimental error appears to be consistent over the span of all three experiments, it does not influence the overall outcome that is discussed below.

FA and SA follow the expected trend of linear decrease of chemical shift (Figures S6 and S7). With the increasing addition of KOH, more COOH groups become deprotonated. Therefore, a negative charge is gradually exerted onto the scaffold of the analyte. This results in a continuous upfield effect (a decrease in chemical shift) that shields the surrounding nuclei, thereby causing their signals to appear at lower chemical shift values. It is, therefore, conclusive that the singly deprotonated acid HA–A^- exhibits a chemical shift that is an arithmetic mean of the chemical shifts of native acid HA–HA and doubly deprotonated acid A^-–A^-.

MA breaks the linearity of the chemical shift progression as shown in Figure S8. More precisely, two separate linear correlations with significantly different slopes can be identified: the first for the transition from HA–HA to HA–A^- state; the second for the subsequent deprotonation of HA–A^- to A^-–A^- state. Thus, the chemical shift of the singly deprotonated HA–A^- state does not correspond to the arithmetic mean of chemical shifts of the HA–HA and A^-–A^- states as in the case of FA and SA. This indicates that the singly deprotonated MA behaves differently from analogous HA–A^- states for FA and SA. Such behaviour, despite not being direct evidence by any means, can be a hint of the proton-sharing effect that has been described in the main manuscript. If a proton is indeed shared between the carboxylates of MA, a partially rigid seven-membered ring is formed (Figure S9a). The exerted ring strain enforces the carbonyl groups of the COOH units to planarize with the double bond of MA. Therefore, the electron density of the double bond is partially transferred to these C=O moieties via the mesomeric effect. This results in magnetic deshielding of the ^1H nuclei on the double bond, which partially increases their chemical shift. In other words, there would be two effects in place influencing the chemical shift of singly deprotonated MA, each acting

in opposite direction: (i) the effect of negative charge that suppresses the chemical shift and (ii) ring strain due to proton sharing that facilitates better conjugation between the C=C and C=O double bonds that results in increased chemical shift. Upon further addition of KOH over 1.0 equiv., the chemical shift starts dropping rapidly. The seven-membered ring formed by the proton sharing is disrupted and, therefore, the only effect dictating the chemical shift of the doubly deprotonated A^{-}–A^{-} state is the increased negative charge. This would explain the discrepancy in the slopes for MA.

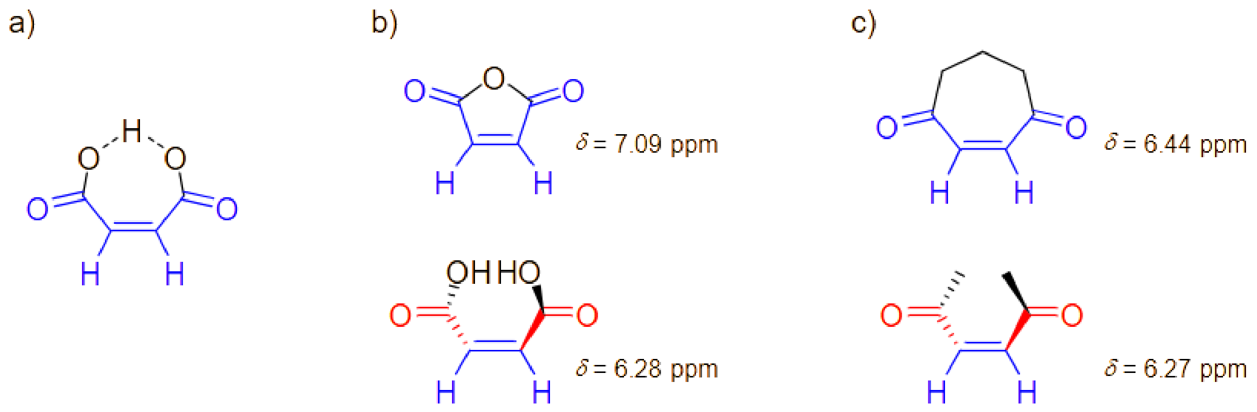

Figure S9: a) proton sharing of MA in HA-A- form; b) maleic anhydride vs. maleic acid: influence of ring strain and resulting mesomeric effect on chemical shift; c) (3Z)-hex-3-ene-2,5-dione vs. cyclohept-2-ene-1,4-dione: influence of ring strain and resulting mesomeric effect.

To support our claim on the ring strain effect exerted by the proton sharing, we show two pairs of structurally similar compounds known from literature for comparison: (i) maleic acid vs. maleic anhydride (Figure S9b),[24] and (ii) (3Z)-hex-3-ene-2,5-dione[25] vs. cyclohept-2-ene-1,4-dione[26] (Figure S9c). The former example shows a dramatic effect on the chemical shift of the double bond signals when the adjacent carbonyls are almost perfectly coplanar with the C=C double bond. The latter case compares a seven-membered ring system with two carbonyls in the vicinity of a C=C double bond that results in a slight increase of the chemical shift as opposed to a similar scaffold that has no ring strain and thus the carbonyl groups are not expected to planarize with the rest of the hydrocarbon backbone due to steric hindrance.

4.2 Vibrational spectroscopies

Femtosecond stimulated Raman spectroscopy (FSRS) is a nonlinear spectroscopic method combining the high spectral resolution of Raman spectroscopy and the high time resolution of femtosecond laser pulses. The question is whether Raman or IR techniques can probe on time scales equal to or shorter than the motion of a proton. In other words, we ask if a vibrational mode can be probed in a temporal interval less than one full vibrational cycle.

The key timescale limitation comes from the nature of the Raman process itself and the probing laser pulses given by the time-bandwidth product in spectroscopy, i.e., the Heisenberg uncertainty principle in quantum mechanics. To achieve a high temporal resolution, the pump as well as the probing pulse must be very short. But even if a femtosecond actinic pump pulse and a femtosecond broadband probe pulse are used in FSRS, a time resolution only as fast as 30–50 fs can be achieved.[24,26,27] This duration is on the order of one vibrational oscillation cycle (or more) to occur. Thus, Raman or IR techniques may not be able to resolve whether a proton is actually delocalized or if it is oscillating or 'hopping' between two neighbouring protonation sites. Furthermore, it cannot be excluded that solvent water molecules assist in the proton exchange. However, vibrational modes of protonation sites should be affected depending on how or to what extent a proton is attached, in a similar way as, for example, a vibrational band shift of a carboxylic group upon deprotonation. Thus, Raman or IR techniques, similarly to NMR, can only bring indirect information on the PS phenomenon.

References

(1) Preobrajenski, A.; Generalov, A.; Öhrwall, G.; Tchaplyguine, M.; Tarawneh, H.; Appelfeller, S.; Frampton, E.; Walsh, N. FlexPES: a versatile soft X-ray beamline at MAX IV Laboratory. *J. Synchrotron Radiat.* **2023**, *30*, 831–840.

(2) Thürmer, S.; Malerz, S.; Trinter, F.; Hergenhahn, U.; Lee, C.; Neumark, D. M.; Mei-

jer, G.; Winter, B.; Wilkinson, I. Accurate vertical ionization energy and work function determinations of liquid water and aqueous solutions. *Chem. Sci.* **2021**, *12*, 10558–10582.

(3) Lide, D. R. *CRC Handbook of Chemistry and Physics*, 73rd ed.; CRC Press, 1992.

(4) Tomaník, L.; Pugini, M.; Mudryk, K.; Thürmer, S.; Stemer, D.; Credidio, B.; Trinter, F.; Winter, B.; Slavíček, P. Liquid-jet photoemission spectroscopy as a structural tool: site-specific acid–base chemistry of vitamin C. *Phys. Chem. Chem. Phys.* **2024**, *26*, 19673–19684.

(5) Mennucci, B.; Tomasi, J. Continuum solvation models: A new approach to the problem of solute's charge distribution and cavity boundaries. *J. Chem. Phys.* **1997**, *106*, 5151–5158.

(6) Cancès, E.; Mennucci, B.; Tomasi, J. A new integral equation formalism for the polarizable continuum model: Theoretical background and applications to isotropic and anisotropic dielectrics. *J. Chem. Phys.* **1997**, *107*, 3032–3041.

(7) Rappe, A. K.; Casewit, C. J.; Colwell, K. S.; Goddard, W. A. I.; Skiff, W. M. UFF, a full periodic table force field for molecular mechanics and molecular dynamics simulations. *J. Am. Chem. Soc.* **1992**, *114*, 10024–10035.

(8) Frisch, M. J. et al. Gaussian 09 Revision D.01. Gaussian Inc. Wallingford CT 2009.

(9) Yanai, T.; Tew, D. P.; Handy, N. C. A new hybrid exchange–correlation functional using the Coulomb-attenuating method (CAM-B3LYP). *Chem. Phys. Lett.* **2004**, *393*, 51–57.

(10) Gilbert, A. T. B.; Besley, N. A.; Gill, P. M. W. Self-Consistent Field Calculations of Excited States Using the Maximum Overlap Method (MOM). *J. Phys. Chem. A* **2008**, *112*, 13164–13171.

(11) Dunning, T. H. Gaussian basis sets for use in correlated molecular calculations. I. The atoms boron through neon and hydrogen. *J. Chem. Phys.* **1989**, *90*, 1007–1023.

(12) Kendall, R. A.; Dunning, T. H.; Harrison, R. J. Electron affinities of the first-row atoms revisited. Systematic basis sets and wave functions. *J. Chem. Phys.* **1992**, *96*, 6796–6806.

(13) Woon, D. E.; Dunning, T. H. Gaussian basis sets for use in correlated molecular calculations. V. Core-valence basis sets for boron through neon. *J. Chem. Phys.* **1995**, *103*, 4572–4585.

(14) Ambroise, M. A.; Jensen, F. Probing Basis Set Requirements for Calculating Core Ionization and Core Excitation Spectroscopy by the Δ Self-Consistent-Field Approach. *J. Chem. Theory Comput.* **2019**, *15*, 325–337.

(15) Cammi, R.; Tomasi, J. Nonequilibrium solvation theory for the polarizable continuum model: A new formulation at the SCF level with application to the case of the frequency-dependent linear electric response function. *Int. J. Quantum Chem.* **1995**, *56*, 465–474.

(16) Herbert, J. M. Dielectric continuum methods for quantum chemistry. *Wiley Interdiscip. Rev. Comput. Mol. Sci.* **2021**, *11*, e1519.

(17) Epifanovsky, E. et al. Software for the frontiers of quantum chemistry: An overview of developments in the Q-Chem 5 package. *J. Chem. Phys.* **2021**, *155*, 084801.

(18) Arabi, A. A.; Matta, C. F. Effects of external electric fields on double proton transfer kinetics in the formic acid dimer. *Phys. Chem. Chem. Phys.* **2011**, *13*, 13738–13748.

(19) Daly, A. M.; Douglass, K. O.; Sarkozy, L. C.; Neill, J. L.; Muckle, M. T.; Zaleski, D. P.; Pate, B. H.; Kukolich, S. G. Microwave measurements of proton tunneling and structural parameters for the propiolic acid–formic acid dimer. *J. Chem. Phys.* **2011**, *135*, 154304.

(20) Evangelisti, L.; Écija, P.; Cocinero, E. J.; Castaño, F.; Lesarri, A.; Caminati, W.; Meyer, R. Proton Tunneling in Heterodimers of Carboxylic Acids: A Rotational Study of the Benzoic Acid–Formic Acid Bimolecule. *J. Phys. Chem. Lett.* **2012**, *3*, 3770–3775.

(21) Feng, G.; Favero, L. B.; Maris, A.; Vigorito, A.; Caminati, W.; Meyer, R. Proton Transfer in Homodimers of Carboxylic Acids: The Rotational Spectrum of the Dimer of Acrylic Acid. *J. Am. Chem. Soc.* **2012**, *134*, 19281–19286.

(22) Zhou, Z.; Aitken, R. A.; Cardinaud, C.; Slawin, A. M. Z.; Wang, H.; Daly, A. M.; Palmer, M. H.; Kukolich, S. G. Synthesis, microwave spectra, x-ray structure, and high-level theoretical calculations for formamidinium formate. *J. Chem. Phys.* **2019**, *150*, 094305.

(23) Liu, H.; Cao, J.; Bian, W. Double Proton Transfer in the Dimer of Formic Acid: An Efficient Quantum Mechanical Scheme. *Front. Chem.* **2019**, *Volume 7 - 2019*.

(24) Abraham, R. J.; Bardsley, B.; Mobli, M.; Smith, R. J. 1H chemical shifts in NMR. Part 21—Prediction of the 1H chemical shifts of molecules containing the ester group: a modelling and ab initio investigation. *Magn. Reson. Chem.* **2005**, *43*, 3–15.

(25) Tönjes, J.; Medvarić, V.; Werner, T. Synthesis of Trisubstituted Furans from Activated Alkenes by P(III)/P(V) Redox Cycling Catalysis. *J. Org. Chem.* **2024**, *89*, 10729–10735.

(26) Kawasumi, M.; Kanoh, N.; Iwabuchi, Y. Concise Entry to Both Enantiomers of 8-Oxabicyclo[3.2.1]oct-3-en-2-one Based on Novel Oxidative Etherification: Formal Synthesis of (+)-Sundiversifolide. *Org. Lett.* **2011**, *13*, 3620–3623.

(27) Lynch, P. G.; Das, A.; Alam, S.; Rich, C. C.; Frontiera, R. R. Mastering Femtosecond Stimulated Raman Spectroscopy: A Practical Guide. *ACS Phys. Chem. Au* **2024**, *4*, 1–18.
